# Automated detection of hyperdense artery sign on non-contrast CT for rapid identification of large vessel occlusion: a multicenter validation study

**DOI:** 10.3389/fneur.2026.1771379

**Published:** 2026-04-20

**Authors:** Hirofumi Tsuji, Akira Ishii, Hidehisa Nishi, Yu Abekura, Takuya Fuchigami, Atsushi Tachibana, Hirotaka Ito, Yoshiki Arakawa

**Affiliations:** 1 Shizuoka General Hospital, Shizuoka, Japan; 2 Juntendo University Hospital, Bunkyo, Japan; 3 Kokura Memorial Hospital, Kitakyushu, Japan; 4 FUJIFILM Corporation, Tokyo, Japan; 5 Kyoto University, Kyoto, Japan

**Keywords:** artificial intelligence, computed tomography, deep learning, hyperdense artery sign, ischemic stroke, large vessel occlusion, workflow triage

## Abstract

**Purpose:**

Computed tomography angiography (CTA) is the gold standard for detecting large vessel occlusion, but its acquisition and reconstruction delay time-critical workflow. The hyperdense artery sign (HAS) on non-contrast CT (NCCT) offers an immediate, albeit subtle, marker. We developed a fully automated deep-learning model for HAS detection and evaluated its utility as an adjunctive pre-CTA alert to support earlier workflow readiness while confirmatory vascular imaging is pending. Furthermore, we assessed the radiological validity of the model’s detections to ensure they correspond to genuine thrombi rather than artifacts.

**Methods:**

We trained a 3-step deep-learning pipeline (midline correction, ischemic core segmentation, HAS segmentation) on 690 NCCT scans. Clinical validation was performed in two complementary cohorts: Part 1A, a multicenter CSC triage cohort (*n* = 159) representing a workflow-enriched high-acuity setting, and Part 1B, a single-center consecutive all-comer suspected-stroke cohort (*n* = 226) representing a broader real-world population. The primary metric was the Positive Predictive Value (PPV) to assess the reliability of the alert as a workflow-support role. Technical validation was performed using a crossover multi-reader study (*n* = 10 specialists and residents) to evaluate whether AI-detected regions were radiologically perceivable by human readers.

**Results:**

In Part 1A, the model achieved a sensitivity of 76.2% (80/105), specificity of 87.0% (47/54), accuracy of 79.9% (127/159), and PPV of 92.0% (80/87), indicating high reliability of positive alerts in a CSC triage setting. In Part 1B, the model achieved a sensitivity of 74.3% (26/35), specificity of 82.7% (158/191), PPV of 44.1% (26/59), NPV of 94.6% (158/167), and accuracy of 81.4% (184/226), reflecting preserved discrimination in a lower-prevalence, broader real-world population. In the reader study, model assistance significantly improved HAS-detection performance, increasing JAFROC Figure of Merit from 0.71 to 0.77 (*p* < 0.01).

**Conclusion:**

The proposed model enables rapid HAS detection on NCCT and demonstrated complementary performance across two validation settings: high reliability of positive alerts in a workflow-enriched CSC triage cohort and preserved sensitivity/specificity in a broader consecutive cohort. These findings support its role as an adjunctive pre-CTA alert for earlier workflow readiness in high-probability settings, not as a stand-alone rule-out tool. The observer study further supports the radiological validity of the AI-highlighted regions.

## Introduction

Large vessel occlusion (LVO) accounts for approximately 30% of acute ischemic stroke (17). As timely recanalization significantly improves patient outcomes, rapid and reliable detection is a critical component of patient care (1). Currently, Computed Tomography Angiography serves as the primary diagnostic modality due to its accuracy and accessibility (2). However, CTA acquisition, reconstruction, and interpretation inevitably add critical minutes to the workflow, especially in primary stroke centers (PSCs) (3). This latency creates a significant bottleneck, delaying angio-suite mobilization in comprehensive stroke centers (CSCs) and extending Door-in-Door-out times in PSCs where transfer decisions depend on confirming occlusion.

The hyperdense artery sign (HAS) on non-contrast CT (NCCT) reflects intraluminal thrombus and can serve as an immediate marker of LVO (4). While the visual sensitivity of HAS is modest (40–65%) for human readers, recent advances in deep learning offer the potential for automated detection (4–6). However, the clinical value of such AI tools should not be to replace CTA or merely assist human interpretation. Instead, considering the time-critical nature of stroke, an automated alert may serve as an adjunctive Pre-CTA alert to support earlier workflow readiness. Such a trigger would allow for a paradigm shift from a serial to a parallel workflow: initiating angio-suite preparation in CSCs, and facilitating rapid “drip-and-ship” decisions in PSCs, potentially before CTA reconstruction is complete.

More broadly, recent deep-learning research in cerebrovascular imaging has increasingly shifted from standalone diagnostic performance toward integration into real-world clinical workflows. In this context, implementation-oriented metrics such as positive predictive value, false-alert burden, and compatibility with physician-supervised decision-making are as important as discrimination performance itself. Recent systematic reviews in intracranial vascular imaging have similarly emphasized that the clinical value of deep-learning systems depends not only on accuracy, but also on how seamlessly they can be incorporated into time-sensitive workflows and whether their outputs can support rather than disrupt clinical operations (7). Our study addresses this translational gap by evaluating automated HAS detection not as a replacement for CTA, but as a workflow-support tool to facilitate earlier awareness and preparatory steps while confirmatory vascular imaging is pending.

In time-critical stroke workflows, automated HAS detection may function as an adjunctive pre-CTA alert that supports earlier parallel workflow readiness while confirmatory vascular imaging is still pending. For such a role to be clinically meaningful, the AI model should satisfy two conditions: first, positive alerts should be sufficiently precise to justify earlier workflow awareness in a consecutive cohort; and second, the detected regions should be radiologically valid, meaning that the model should respond to genuine thrombus-related image features rather than nonspecific artifacts or noise.

We therefore (1) developed a fully automated deep-learning pipeline for HAS detection, (2) evaluated it in two complementary clinical validation cohorts: Part 1A, a multicenter CSC triage cohort representing a workflow-enriched high-acuity setting, and Part 1B, a single-center consecutive suspected-stroke cohort representing a broader real-world screening population, and (3) conducted a multi-reader observer study to test whether the model-localized findings correspond to radiologically perceivable thrombus signs that can augment human detection.

## Methods

### Study design and strategy

This multicenter retrospective study was approved by the institutional review boards of all participating centers. The study consisted of four components: (1) a derivation cohort for model development, (2) Part 1A, a multicenter CSC-enriched clinical validation cohort designed to evaluate the model in a workflow-oriented high-acuity triage setting, (3) Part 1B, a single-center fully consecutive clinical validation cohort designed to evaluate performance in a broader real-world suspected-stroke population, and (4) a crossover multi-reader observer study designed to assess the radiological validity of the AI-localized findings.

The clinical validation component was therefore performed in two complementary settings. Part 1A was intended to assess the reliability of positive alerts in a CSC-oriented triage context, where the pretest probability of anterior circulation LVO is relatively high. Part 1B was intended to assess the model’s discrimination performance in a lower-prevalence, fully consecutive population that more closely reflects an unfiltered real-world screening setting. The observer study served as technical validation to determine whether AI-highlighted regions corresponded to radiologically perceivable thrombus-related image features rather than nonspecific artifacts.

### Derivation cohort (model training)

We collected 690 NCCT scans from four stroke centers (Kobe City Medical Center General Hospital, Kokura Memorial Hospital, Kyoto University Hospital, and Saiseikai Kumamoto Hospital) to train the deep learning pipeline.

### Image acquisition, model architecture, and training configuration

NCCT images were acquired on eight different scanner models from five venders (Philips, Siemens, Canon, GE) with 120 kVp, 250–440 mA, either in helical or axial mode, and 1-mm in-plane resolution reconstructed at 3.0–8.0 mm slice thickness. Images were resampled to 0.75 × 0.75 mm in-plane; native slice thickness was preserved.

**Figure 1:**
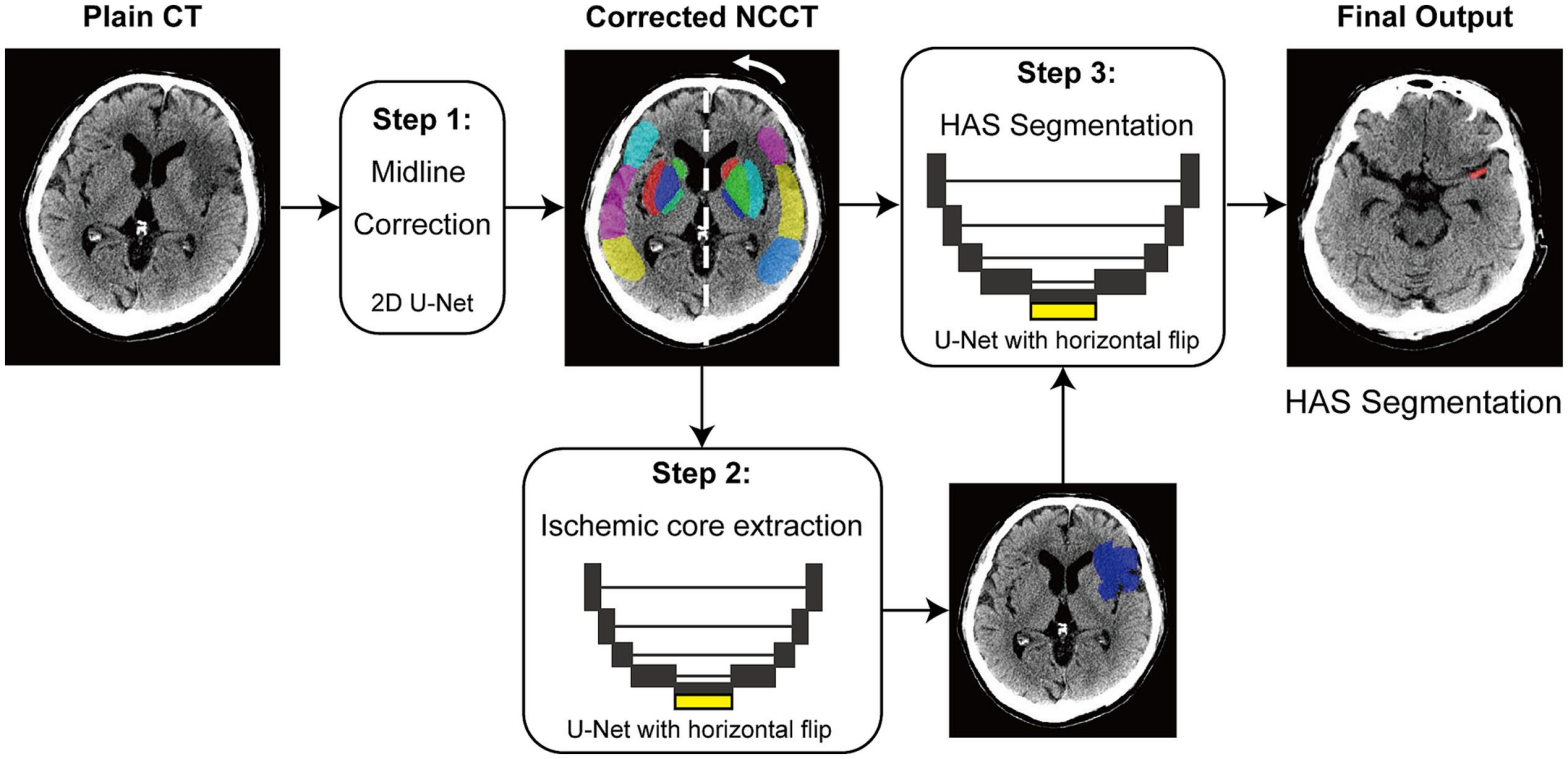
Automated pipeline for core-guided HAS detection. The system processes non-contrast CT (NCCT) images in three stages to generate an adjunctive pre-CTA alert. (Step 1) Image alignment: The input NCCT undergoes automated midline correction and normalization to standardize anatomical positioning. (Step 2) Ischemic core extraction: A hemisphere-comparison network identifies the ischemic core (blue overlay), creating a context map to suppress false positives from chronic lesions or artifacts. (Step 3) Core-guided segmentation: A 3D U-Net segments the hyperdense artery sign (HAS, red overlay) using the aligned NCCT, conditioned by the ischemic core map as an attention mechanism. Output: The system generates a high-precision alert (PPV 92.0%) within 5 s, which may support earlier workflow readiness and team notification while confirmatory vascular imaging is pending.

The automated pipeline consists of three sequential stages: (1) midline correction via a 2D U-Net for anatomical standardization, (2) ischemic core extraction using a 3D hemisphere-comparison network as detailed in our previous report (8, 9), and (3) core-guided HAS segmentation via a 3D U-Net. A distinct feature of this architecture is the utilization of the ischemic core map (from Stage 2) as an attention mechanism for the final segmentation (Figure 1). By inputting the core map alongside the corrected NCCT image, the model is designed to prioritize vascular hyperdensities spatially associated with early ischemic changes, thereby minimizing false positives derived from mimics such as calcifications or image noise.

For training of the final HAS-segmentation stage, the network received full-volume inputs and was optimized using cross-entropy loss with the Adam optimizer (learning rate, 0.0015; batch size, 4). Training was performed for 440 epochs without early stopping. Data augmentation included random rotation, translation, scaling, Gaussian noise addition, and Gaussian blurring. The model was implemented in TensorFlow and trained on an NVIDIA Tesla V100 GPU. A fixed random seed was not enforced during training. Of the 690 derivation CT scans, 60 (approximately 10%) were randomly selected as an internal validation set for model selection and hyperparameter tuning, and the remaining scans were used for training. No separate internal test set was used.

### Ground truth annotation for HAS segmentation

For the derivation cohort, expert-defined reference masks of the hyperdense artery sign (HAS) were created on NCCT using the research-use mask creation tool implemented in VINCENT (FUJIFILM, Tokyo, Japan). Two neuroendovascular physicians manually delineated visually appreciable intravascular hyperattenuation considered compatible with HAS, with reference to the occlusion site confirmed on CTA or MRA. Image co-registration between NCCT and CTA/MRA was not performed; instead, the vascular occlusion site identified on CTA/MRA was visually correlated with the corresponding arterial segment on NCCT. Potential mimics, particularly vascular calcifications, were excluded by reviewing the bone window. Because the annotations were generated in an interactive consensus-based workflow rather than as fully independent duplicate segmentations, formal voxel-wise inter-rater agreement was not assessed. In cases of disagreement regarding the presence or extent of HAS, the final reference mask was determined by consensus between the two annotators.

#### Part 1A: multicenter CSC-enriched triage cohort (clinical validation)

Part 1A was designed to evaluate the model’s utility as an adjunctive pre-CTA alert in a workflow-enriched CSC triage setting. We collected a multicenter cohort of 159 patients (105 confirmed LVOs and 54 controls) from five stroke centers (Kobe City Medical Center General Hospital, Kyoto Medical Center, Kyoto University Hospital, Rakuwakai Otowakinen Hospital, and Koseikai Takeda Hospital). This cohort was designed to reflect the workflow of comprehensive stroke centers, where the prevalence of LVO is relatively high because of emergency transport prioritization and interhospital referrals.

For the LVO group, inclusion criteria were: (1) anterior circulation LVO (intracranial ICA, M1, or M2) confirmed by CTA or MRA; (2) baseline NCCT acquired within 24 h of onset; and (3) age ≥18 years. Inclusion in the LVO group was based on vascular imaging confirmation rather than visual HAS positivity on NCCT; therefore, LVO cases without visually appreciable HAS were not excluded from the consecutive validation cohort. The control group consisted of patients evaluated for acute focal neurologic deficits who were diagnosed with non-LVO ischemic stroke, intracranial hemorrhage, or stroke mimics.

#### Part 1B: single-center fully consecutive all-comer cohort (clinical validation)

Part 1B was designed to assess model performance in a broader real-world population. We assembled a single-center fully consecutive cohort of 226 patients who underwent NCCT as part of the diagnostic workup for suspected stroke. This cohort was intended to represent a lower-prevalence, less enriched clinical environment than Part 1A.

Ground-truth LVO status in Part 1B was determined based on final vascular imaging adjudication. The target condition was anterior circulation LVO. Unlike Part 1A, which was designed to reflect CSC-oriented triage conditions, Part 1B was designed to evaluate model discrimination in a broader consecutive population, including ischemic stroke, intracranial hemorrhage, subarachnoid hemorrhage, and other stroke-mimicking or non-LVO conditions encountered in routine emergency practice.

#### Part 2: crossover observer study (technical validation)

To verify the radiological validity of the model’s output, we conducted a multi-reader crossover study using an enriched dataset derived from Part 1A. This sub-study was designed to assess radiological conspicuity rather than screening performance. The final observer dataset consisted of 129 cases, including 76 LVO-positive cases with visually appreciable HAS and 53 LVO-negative/HAS-negative control cases. Cases in which HAS was not visually appreciable to human readers were excluded because the purpose of this sub-study was to evaluate whether AI assistance could improve recognition of potentially perceivable thrombus signs rather than detect completely occult lesions. Ten readers—comprising five board-certified neurointerventionalists (>9 years of experience) and five residents (≤3 years)—interpreted scans with and without model assistance. Case order was randomized for each session, readers were blinded to the reference diagnosis and case composition, and a 4-week washout period was applied between sessions.

### Statistical analysis

For Part 1A and Part 1B, classification metrics (sensitivity, specificity, PPV, NPV) were calculated with 95% confidence intervals. Since the model provided binary outputs, we calculated balanced accuracy (mean of sensitivity and specificity) instead of the area under the ROC curve. Site-specific sensitivity (ICA vs. M1 vs. M2) was also analyzed.

For the observer study (Technical Validation), we evaluated the change in diagnostic accuracy using the area under the receiver operating characteristic curve (AUC) and figure of merit (FOM) from Jackknife Free-Response ROC (JAFROC) analysis (10). Improvement in AUC and FOM was interpreted as evidence of the model’s capability to correctly localize radiologically valid thrombi. Statistical significance was defined as *p* < 0.05.

## Results

### Baseline characteristics

**Figure 2:**
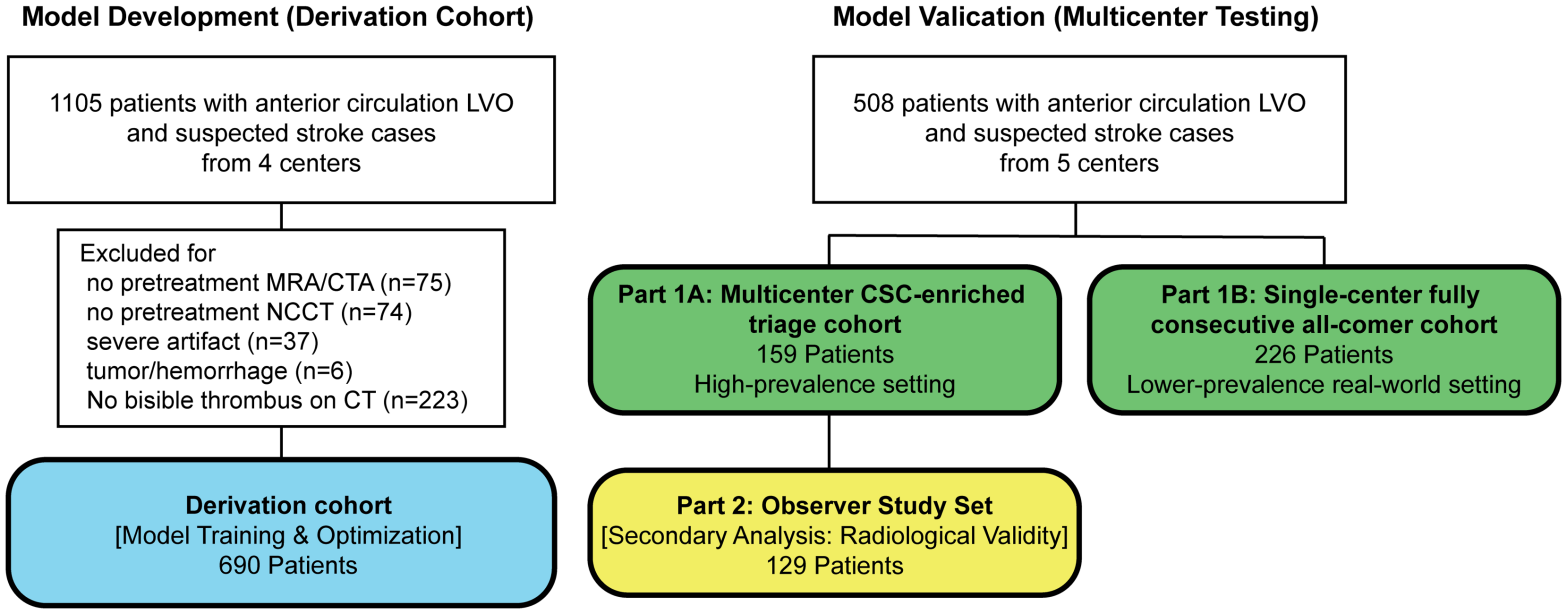
Patient flow diagram. The study consisted of a derivation cohort for model training (*n* = 690), two complementary clinical validation cohorts, and a technical validation observer study. Part 1A comprised a multicenter CSC-enriched triage cohort (*n* = 159) designed to assess the reliability of positive AI alerts in a high-acuity workflow setting. Part 1B comprised a single-center fully consecutive all-comer cohort (*n* = 226) designed to assess model performance in a broader real-world population. Part 2 was a crossover multi-reader observer study (*n* = 129), derived from Part 1A, to evaluate the radiological conspicuity of AI-localized findings.

The patient flowchart is shown in Figure 2. The baseline characteristics of the derivation cohort, Part 1A cohort, and Part 1B cohort are summarized in Table 1. Diagnostic performance in the two clinical validation cohorts is summarized in Table 2.

**Table 1:** Patient characteristics of each cohort.

Characteristic	Derivation cohort (*n* = 690)	Part 1A CSC-enriched triage cohort (*n* = 159)	Part 1B fully consecutive all-comer cohort (*n* = 226)
Age, years			
Mean ± SD	72.1 ± 14.1	75.0 ± 13.5	74.2 ± 13.0
Median (IQR)	74.0 (64–82)	78.0 (68–82)	76.0 (67–84)
Male sex, n (%)	163/286 (57.0)	78/149 (52.3)	115/226 (50.9)
LVO-positive, n (%)	228 (33.0)	105 (66.0)	35 (15.5)
LVO site			
ICA, n (%)	68 (29.8)	33 (31.4)	5 (14.3)
MCA M1, n (%)	95 (41.7)	53 (50.5)	18 (51.4)
MCA M2, n (%)	65 (28.5)	17 (16.2)	10 (28.6)
Other, n (%)	1 (0.4)	1 (1.0)	2 (5.7)
Control group, n (%)	462 (67.0)	54 (34.0)	191 (84.5)

ICA, internal carotid artery; LVO, large-vessel occlusion; SD, standard deviation. Percentages for sex were calculated using available cases only because sex data were missing for 404 patients in the derivation cohort and 10 patients in Part 1A due to site-specific anonymization procedures; no sex data were missing in Part 1B.

**Table 2:** Diagnostic performance in the validation cohorts.

Cohort	n	Sensitivity (95%CI)	Specificity (95%CI)	PPV (95%CI)	NPV (95%CI)	Accuracy (95%CI)
Part 1A CSC-enriched triage cohort	159	76.2% (66.9–84.0)	87.0% (75.1–94.6)	92.0% (84.1–96.7)	65.3% (53.1–76.1)	79.9% (72.8–85.8%)
Part 1B fully consecutive all-comer cohort	226	74.3% (56.7–87.5)	82.7% (76.6–87.8)	44.1% (31.2–57.6)	94.6% (90.0–97.5)	81.4% (75.7–86.3)

CI, confidence interval; PPV, positive predictive value; NPV, negative predictive value.

The derivation cohort included 690 NCCT scans collected from four stroke centers. Part 1A consisted of 159 patients from five stroke centers, including 105 anterior circulation LVO cases and 54 controls, representing a CSC-enriched triage population. Part 1B consisted of 226 fully consecutive patients from a single center, representing a broader all-comer suspected-stroke population. In Part 1B, the prevalence of target LVO was 15.5% (35/226), substantially lower than that in Part 1A (66.0%, 105/159), consistent with the different intended roles of the two clinical validation cohorts.

Sex data were unavailable for 404 of 690 patients in the derivation cohort and for 10 of 159 patients in Part 1A because anonymization procedures at participating centers resulted in loss of sex metadata.

#### Part 1A: clinical utility in a CSC-enriched triage cohort

**Figure 3:**
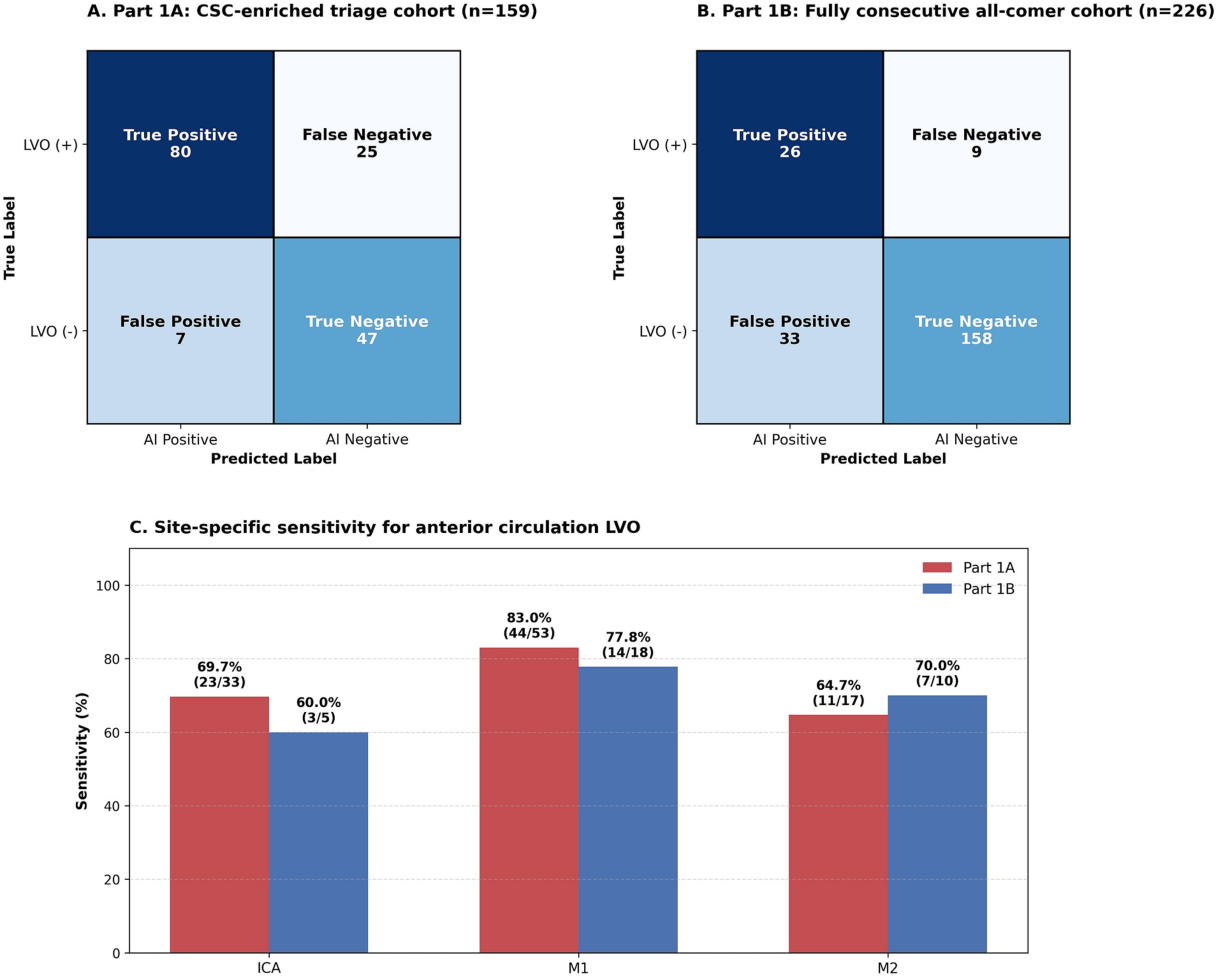
Performance in the consecutive triage cohort. **(A)** Confusion matrix for part 1A, the multicenter CSC-enriched triage cohort (*n* = 159). In this high-prevalence setting, the model achieved a PPV of 92.0%, indicating high reliability of positive alerts. **(B)** Confusion matrix for part 1B, the single-center fully consecutive all-comer cohort (*n* = 226). In this broader lower-prevalence setting, the model maintained sensitivity and specificity, while PPV decreased because of the lower prevalence of LVO. **(C)** Site-specific sensitivity analysis for anterior circulation LVO, comparing ICA, M1, and M2 occlusions across part 1A and part 1B. ICA, internal carotid artery; M1, M1 segment of the middle cerebral artery; M2, M2 segment of the middle cerebral artery.

The confusion matrix for Part 1A is shown in Figure 3. In this multicenter CSC-enriched triage cohort, the model achieved a PPV of 92.0% (80/87), indicating that positive AI alerts were highly associated with true anterior circulation LVO in this high-probability setting. The sensitivity was 76.2% (80/105), specificity was 87.0% (47/54), NPV was 65.3% (47/72), and accuracy was 79.9% (127/159). For reference, applying the Part 1A sensitivity (76.2%) and specificity (87.0%) to a hypothetical setting with 30% LVO prevalence yields an estimated PPV of 71.5% and NPV of 89.5%.

Site-specific analysis showed the highest sensitivity of 83.0% (44/53) for M1 occlusions, followed by ICA occlusions at 69.7% (23/33) and M2 occlusions at 64.7% (11/17). Because Part 1A was designed to reflect CSC triage conditions rather than an unselected emergency department screening population, these findings support the reliability of positive alerts as an adjunctive pre-CTA workflow signal in selected high-probability settings.

**Figure 4:**
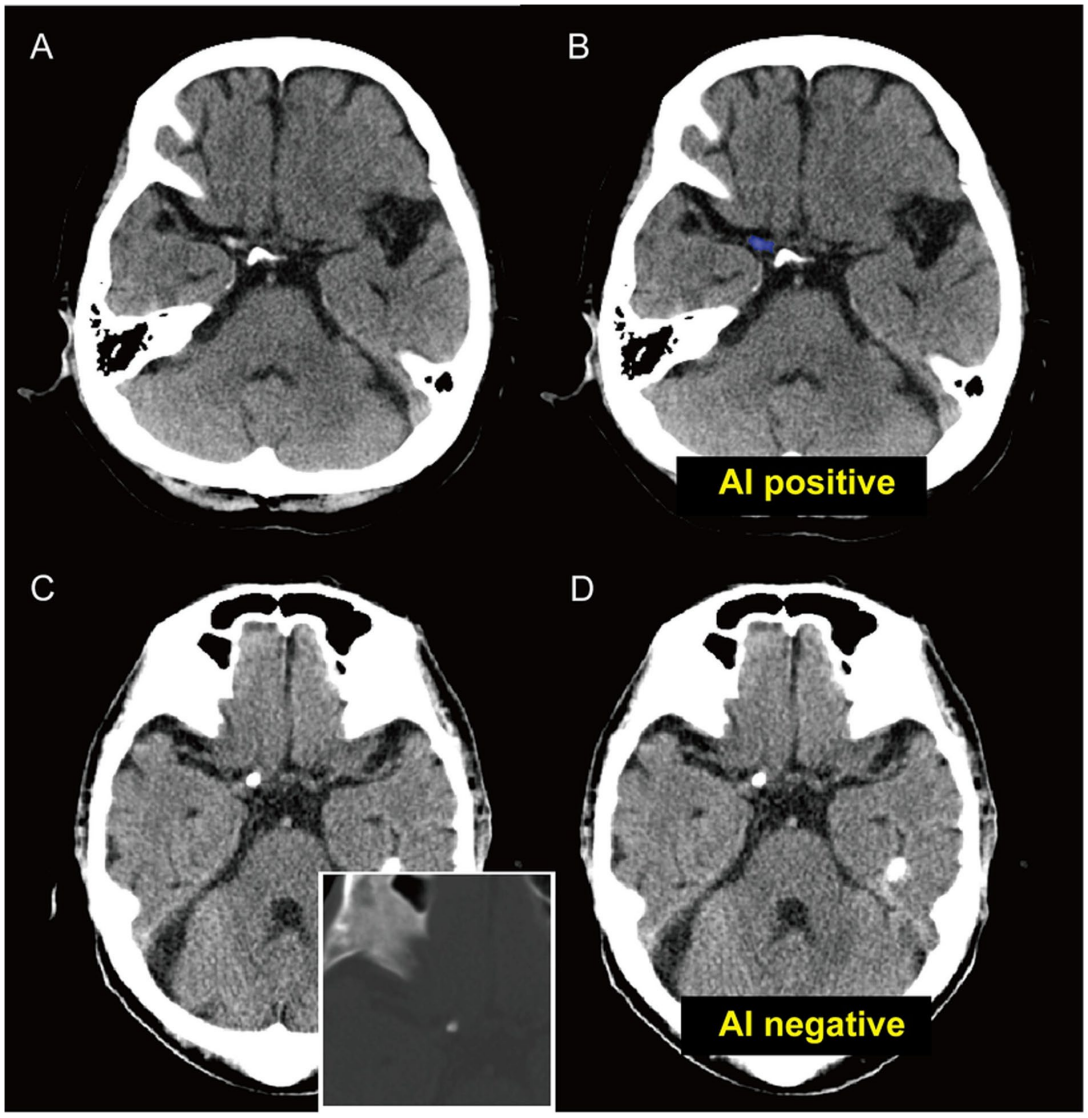
Representative cases illustrating the potential role of the AI model as an adjunctive pre-CTA alert tool. **(A,B)** A subtle hyperdense artery sign (HAS) in the ICA **(A)**. The AI model correctly localized the lesion (blue overlay), demonstrating its ability to identify subtle HAS findings that may support earlier workflow awareness while confirmatory vascular imaging is pending **(B)**. **(C,D)** Reliability against mimics: A case with intracranial calcification mimicking HAS. The model correctly rejected this region **(D)**, illustrating specificity against common mimics and supporting the reliability of positive alerts in this study setting.

Importantly, Part 1A was not restricted to visually appreciable HAS-positive cases. Among 30 CTA/MRA-confirmed LVO cases without visually appreciable HAS on NCCT, the model generated positive alerts in 18 cases (60.0%). Other performance metrics are described in Table 2. The representative cases are shown in Figure 4.

#### Part 1B: performance in a fully consecutive all-comer cohort

In the fully consecutive Part 1B cohort (*n* = 226), the model yielded a sensitivity of 74.3% (26/35), specificity of 82.7% (158/191), PPV of 44.1% (26/59), NPV of 94.6% (158/167), and accuracy of 81.4% (184/226).

Site-specific sensitivity in Part 1B was 60.0% (3/5) for ICA occlusions, 77.8% (14/18) for M1 occlusions, and 70.0% (7/10) for M2 occlusions. In contrast to Part 1A, the lower PPV in Part 1B should be interpreted in the context of the substantially lower LVO prevalence in this broader real-world population. Nevertheless, the model maintained reasonable sensitivity and specificity, supporting its discrimination performance in a fully consecutive, lower-prevalence screening setting.

Exploratory sex-stratified analysis in Part 1B showed similar specificity between male and female patients (81.0% vs. 84.9%), whereas sensitivity was numerically higher in males (90.0% vs. 68.0%). However, subgroup sample sizes were limited, and predictive values appeared to be influenced by the difference in LVO prevalence between the two groups (8.7% vs. 22.5%).

Taken together, the two clinical validation cohorts provided complementary perspectives: Part 1A demonstrated high reliability of positive alerts in a workflow-enriched CSC triage context, whereas Part 1B demonstrated maintained sensitivity and specificity in a broader unfiltered real-world population.

#### Part 2: technical validation of radiological conspicuity (observer study)

To verify the radiological validity of the model’s detections, we analyzed the change in human reader performance (*n* = 129 observations) on an enriched dataset of visible thrombi. The baseline diagnostic accuracy of human readers without AI assistance was moderate, with a mean AUC of 0.72. Model assistance significantly improved the mean AUC to 0.81 (*Δ* + 0.09, *p* < 0.01).

**Figure 5:**
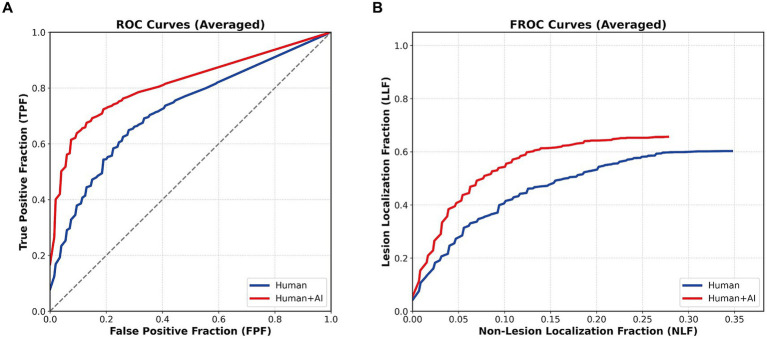
Impact of model assistance on reader performance (multi-reader study). **(A)** ROC curves: Diagnostic accuracy of 10 readers (specialists and residents) for detecting HAS. The curve for model-assisted reading (red: Human+AI) is shifted to the upper left compared to unassisted reading (blue: Human), demonstrating a significant improvement in diagnostic accuracy (mean AUC increased from 0.72 to 0.81, *p* < 0.01). **(B)** FROC curves: Localization performance showing lesion localization fraction (LLF) versus non-lesion localization fraction (NLF). Model assistance significantly improved the JAFROC figure of merit (FOM) from 0.71 to 0.77 (*p* < 0.01). This improvement confirms that the model accurately localizes radiologically valid thrombus signals perceivable by human readers.

This improvement was consistent across experience levels: board-certified neurointerventionalists improved from 0.71 to 0.83 (*p* < 0.01), and trainees improved from 0.73 to 0.80 (*p* = 0.02). The Figure of Merit (FOM) also showed a significant increase from 0.71 to 0.77 (*p* < 0.01; Figure 5). These findings support the radiological validity of the AI-highlighted regions and indicate that the model localizes thrombus-related image features that are perceivable by human readers when appropriately prompted.

## Discussion

We developed and validated a fully automated deep-learning model for HAS detection on routine NCCT and evaluated it in two complementary clinical settings. In Part 1A, a multicenter CSC-enriched triage cohort, the model achieved a PPV of 92.0% with a sensitivity of 76.2%, supporting the reliability of positive alerts in a high-probability workflow setting. In Part 1B, a fully consecutive all-comer cohort with substantially lower LVO prevalence (15.5%), the model yielded a sensitivity of 74.3% and specificity of 82.7%, indicating that its discrimination performance was reasonably preserved in a broader real-world population. In addition, the observer study showed that AI assistance significantly improved human reader performance, supporting the radiological validity of the AI-localized findings.

The main clinical implication of this study is not that AI should replace CTA or physician judgment, but that rapid NCCT-based HAS detection may support earlier parallel workflow readiness while confirmatory vascular imaging is still being acquired or reconstructed. In a standard serial workflow, angio-suite preparation is typically delayed until CTA acquisition and interpretation, which can add 10–60 min to the door-to-puncture time (3, 11, 12). In selected high-probability settings such as Part 1A, our high PPV suggests that a positive AI alert on NCCT may justify earlier team awareness and preparatory steps while confirmatory vascular imaging is still pending. This concept may be particularly relevant in comprehensive stroke centers, and could also have implications for transfer workflows in primary stroke centers. However, any such use should remain adjunctive and physician-supervised, because final escalation and treatment decisions must still rely on CTA and the full clinical context.

An important strength of the present study is that the model was not assessed only in an enriched triage cohort. Because PPV is inherently prevalence-dependent, exclusive reliance on Part 1A would have left uncertainty regarding generalizability to broader emergency populations. Part 1B directly addressed this issue by evaluating the model in a fully consecutive all-comer cohort. As expected, PPV decreased to 44.1% in this lower-prevalence setting, but sensitivity and specificity remained at 74.3 and 82.7%, respectively. This pattern suggests that the core discriminative capacity of the model was retained, while its practical interpretation changed according to context. In other words, in CSC-enriched high-prevalence settings, the model may function as a reliable adjunctive pre-CTA alert for early workflow preparation, whereas in broader consecutive populations, it should be interpreted more conservatively as a supportive detection tool rather than a stand-alone mobilization trigger. Importantly, despite the high NPV observed in Part 1B, a negative AI result must not be used to exclude LVO or defer CTA-based vascular assessment.

The site-specific findings also merit comment. In Part 1A, sensitivity was highest for M1 occlusions, whereas ICA and M2 occlusions showed lower detectability; a similar pattern was observed in Part 1B. For ICA occlusions, detection may be limited by the complex skull-base and carotid siphon anatomy, where adjacent bone, beam-hardening, and occasional calcified segments can reduce the conspicuity of true intraluminal hyperattenuation on NCCT. For M2 occlusions, the smaller vessel caliber, lower thrombus burden, and greater susceptibility to partial-volume effects likely contribute to reduced detectability on routine clinical CT. At the same time, review of false-positive cases suggested that many false alerts were related not to obvious calcification or motion artifacts, but to subtle intravascular hyperattenuation mimicking HAS. This observation suggests that the model is responding primarily to thrombus-like luminal density changes, while also highlighting the intrinsic difficulty of separating true HAS from borderline vascular hyperattenuation on NCCT. Because more than 90% of scans were reconstructed at 5 mm, meaningful subgroup analysis by slice thickness was not feasible.

Another relevant finding is that the model was not restricted to visually obvious HAS-positive cases. In Part 1A, among 30 CTA/MRA-confirmed LVO cases without visually appreciable HAS on NCCT, the model still generated positive alerts in 18 cases (60.0%). This suggests that the system may capture a subset of subtle thrombus-related imaging patterns that are not readily recognized on routine visual inspection. At the same time, 12 of these 30 cases remained undetected, reinforcing that the current model should not be interpreted as a rule-out system and that CTA remains indispensable for definitive vascular assessment.

Several AI systems for LVO or HAS detection on NCCT have previously been reported. For instance, Olive-Gadea et al. (13) reported a sensitivity of 83% and specificity of 71% using *MethinksLVO*, noting that the addition of NIHSS scores was necessary to improve specificity to 85%. Similarly, Weyland et al. (14) demonstrated that the *Brainomix* software achieved detection performance comparable to specialized physicians (sensitivity 77%, specificity 87%). More recently, Kim et al. (15) reported a robust AUC of 0.888 using handcrafted features in an external validation cohort. We do not claim categorical superiority in raw diagnostic accuracy over all previous approaches. Rather, the distinctive contribution of the present study lies in its workflow-oriented validation design. First, we evaluated the system in a CSC-enriched cohort specifically to assess the reliability of positive alerts in a clinically meaningful pre-CTA triage context. Second, we complemented this with a fully consecutive all-comer cohort to address broader real-world applicability. Third, we performed an observer study to test whether AI-highlighted regions corresponded to radiologically perceivable thrombus features. Together, these components move beyond conventional accuracy reporting and address how such a model might realistically be interpreted and used in practice.

The observer study provides an additional level of clinical relevance. While Weyland et al. positioned AI in comparison to physicians to prove non-inferiority, and Kim et al. (16) highlighted the inherent subjectivity of HAS detection with a moderate inter-expert kappa of 0.521, we demonstrated that model assistance significantly improved the readers’ diagnostic accuracy. Model assistance significantly improved both AUC and JAFROC Figure of Merit, indicating that the AI output was not merely statistically associated with LVO, but also localized image findings that human readers could recognize when appropriately prompted. This supports the radiological plausibility of the model’s detections and suggests that the core-guided architecture enhances practical interpretability. These findings should not be taken as evidence of full explainability of the underlying neural network; rather, they indicate that the model localizes thrombus-related features that are at least partly aligned with human radiological perception.

Our study has several limitations. First, it was retrospective, and prospective implementation studies are needed to determine the true effect of AI alerts on workflow metrics such as time to team notification, angio-suite readiness, transfer initiation, and door-to-puncture time. Second, the derivation labels were created retrospectively on NCCT with visual reference to CTA/MRA-confirmed occlusion sites, and image co-registration was not performed; therefore, some degree of annotation imprecision is possible despite consensus review by two neuroendovascular physicians. Third, sex data were missing for a substantial proportion of patients in the derivation cohort, which limited our ability to fully assess potential demographic bias, although sex was not used as an input for model training. Exploratory sex-stratified analysis in Part 1B did not show a consistent major performance imbalance between male and female patients, although subgroup sample sizes were limited and these findings should be interpreted cautiously. Fourth, the observer study used an enriched dataset focused on potentially visible thrombi and excluded cases without visually appreciable HAS; therefore, those results should be interpreted as evidence of radiological conspicuity rather than screening performance in an unselected population. Fifth, although Part 1B broadened the validation framework substantially, it remained a single-center cohort, and additional prospective multicenter validation in broader suspected-stroke populations is still required. Finally, the present model was developed for anterior circulation occlusions and is intended only as an adjunctive pre-CTA alert; it does not replace CTA or physician-led decision-making for treatment selection.

## Conclusion

The proposed deep-learning model enables rapid detection of the hyperdense artery sign on NCCT and demonstrated complementary performance across two clinical validation settings: high reliability of positive alerts in a CSC-enriched triage cohort and reasonable discrimination in a fully consecutive all-comer cohort. Rather than functioning as an autonomous diagnostic or treatment-triage system, its intended role is as an adjunctive pre-CTA alert that may support earlier workflow readiness and team awareness in selected high-probability anterior circulation stroke settings, while confirmatory vascular imaging remains essential. The observer study further suggests that the model localizes radiologically perceivable thrombus-related features that can augment human detection. Prospective multicenter studies in broader real-world populations are warranted to define its implementation value more precisely.

## Data Availability

The data supporting the conclusions of this article will be made available by the authors upon reasonable request, subject to institutional and ethical regulations.
